# Multiple gastric tumors in patients with combined autoimmune and helicobacter pylori-associated gastritis: a case report and literature review

**DOI:** 10.3389/fimmu.2025.1562853

**Published:** 2025-05-20

**Authors:** Wulian Lin, Cuiling Wu, Guilin Xu, Gaocheng Yi, Guanpo Zhang, Haitao Li, Jin Zheng, Dazhou Li, Wen Wang

**Affiliations:** ^1^ Department of Gastroenterology, 900th Hospital of PLA Joint Logistic Support Force, Fuzhou, Fujian, China; ^2^ Fuzong Clinical Medical College of Fujian Medical University, Fuzhou, Fujian, China; ^3^ Department of Pathology, 900th Hospital of PLA Joint Logistic Support Force, Fuzhou, Fujian, China

**Keywords:** autoimmune gastritis, gastric cancer, endoscopy, helicobacter pylori, case report

## Abstract

This paper presents four cases of autoimmune gastritis coexisting with Helicobacter pylori- associated gastritis, all of which developed gastric tumors, with some cases exhibiting multiple histological origins. Through a narrative literature review, we explore the potential mechanisms and clinical implications of tumor development in this context. This report emphasizes the significance of recognizing synergistic mucosal injury in chronic gastritis management and highlights the need for future studies into combined pathogenesis.

## 1 Introduction

The widely adopted Updated Sydney System classifies chronic atrophic gastritis (CAG) into two major subtypes based on the location of lesions: autoimmune gastritis (AIG) and multifocal atrophic gastritis (MAG) (1). AIG is an organ-specific autoimmune disorder, with or without associated pernicious anemia, mediated by autoantibodies targeting H+-K+-ATPase, specifically parietal cell antibodies (PCA), which selectively lead to the destruction of parietal cells. According to a retrospective study in China, the estimated crude annual detection rate of AIG is approximately 0.9% (2). In contrast, MAG constitutes a significant proportion of chronic gastritis cases in China. Helicobacter pylori (H. pylori) infection is currently recognized as its predominant etiology. With the carcinogenic potential of H. pylori gaining increasing recognition (3), the 2015 Kyoto Global Consensus proposed a classification of gastritis based on etiological factors into three primary categories: H. pylori-associated gastritis, drug-induced gastritis, and AIG (4).

Gastric cancer is the fourth leading cause of cancer-related death worldwide (5) with gastric adenocarcinoma accounting for over 90% of cases. Its development is multifactorial, but H. pylori infection remains the most significant risk factor (6). AIG, a chronic autoimmune inflammatory syndrome, is characterized by autoreactive immune responses against gastric epithelial self-antigens and has been strongly associated with the development of neuroendocrine tumors (NETs) (7). Although the association between AIG and tumorigenesis has been reported, the synergistic carcinogenic mechanisms in the context of coexisting AIG and H. pylori-associated gastritis remain unclear.

Reports of AIG coexisting with H. pylori-associated gastritis are rare, and it remains unclear whether this mucosal context increases tumorigenesis risk. Here, we report four cases of multifocal tumors (including collision tumors) arising in patients with combined AIG and H. pylori-associated gastritis, highlighting the potential for chronic inflammatory synergy to drive heterogeneous neoplasia.

## 2 Case report

### 2.1 Case 1

**Figure 1:**
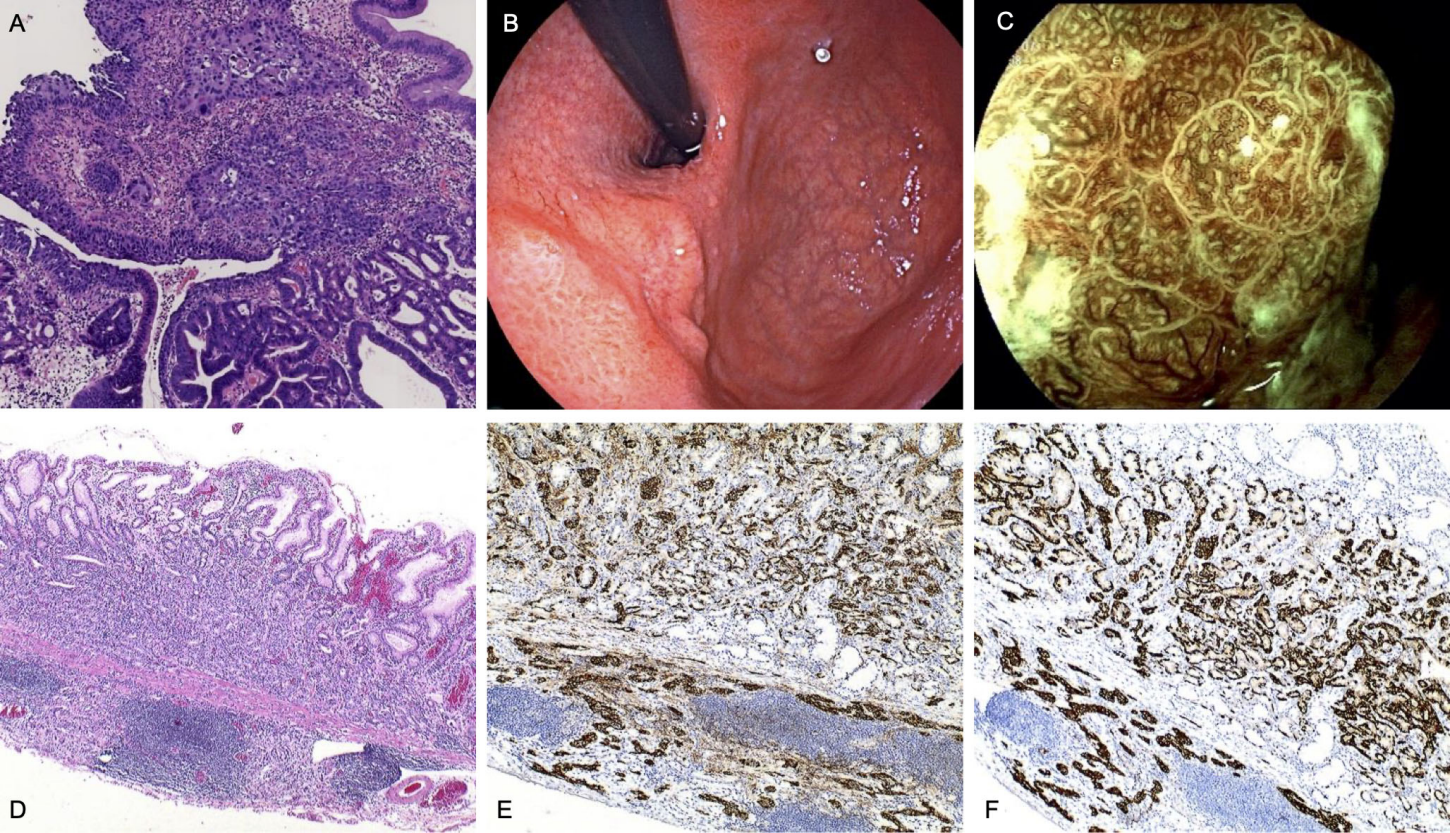
Endoscopic and pathological findings in Case 1. **(A)** Pathology shows adenocarcinoma and squamous cell carcinoma in the polypoid-like lesion. (HE,×4) **(B)** Subcardiac elevated lesion with depressed surface and scar-like changes visible in the center (possibly from previous hospital resection). **(C)** Magnified view of the subcardiac lesion. **(D)** the NET (G1) region:The tumor cells are well-differentiated, with abundant eosinophilic cytoplasm. The nuclei are round, uniform in shape, and lack prominent nucleoli. The morphology is monotonous, and mitotic figures are rare. (HE,×4). **(E)** Immunohistochemical staining for CgA of the NET (G1) region(×10). **(F)** Immunohistochemical staining for Syn of the NET (G1) region(×10).

A 51-year-old woman underwent endoscopic mucosal resection (EMR) for a subcardiac elevated lesion with polypoid features. Histopathological examination revealed a hyperplastic polyp with areas of adenoepithelial high-grade intraepithelial neoplasia and moderately differentiated tubular adenocarcinoma. Additionally, focal squamous epithelial dysplasia was noted at the polyp margin (**Figure 1A**). Following referral to our hospital, magnifying staining gastroscopy revealed severe gastric mucosal atrophy extending to the greater curvature of the corpus (8). Multiple greyish-white flattened elevations were observed in the antrum, with visible light blue crests under magnification. The subcardiac lesion (**Figure 1B**) exhibited poorly defined borders, widened and disordered intervening parts (IP), microvascular dilation, and dark green thickened blood vessels (**Figure 1C**). The patient underwent endoscopic submucosal dissection (ESD), and histopathology confirmed H. pylori infection, a grade 1 neuroendocrine tumor (NET) with submucosal invasion of 400 μm, and an oxyntic gland adenoma (**Figures 1D–F**).

Laboratory tests showed normal hemoglobin, serum iron, folic acid, and vitamin B12, with no evidence of anemia. Serum gastrin levels were within the normal range, while pepsinogen I (PG-I) levels and the PG-I/PG-II ratio were markedly decreased. Tests for intrinsic factor antibodies(IFA) were negative, while PCA were positive. The patient had a history of autoimmune thyroiditis, supporting the diagnosis of AIG.

Based on these findings, the patient was diagnosed with a rare collision tumor comprising adenocarcinoma, squamous carcinoma, oxyntic gland adenoma, and NET. During postoperative follow-up, no evidence of tumor recurrence or anemia was observed.

### 2.2 Case 2

**Figure 2:**
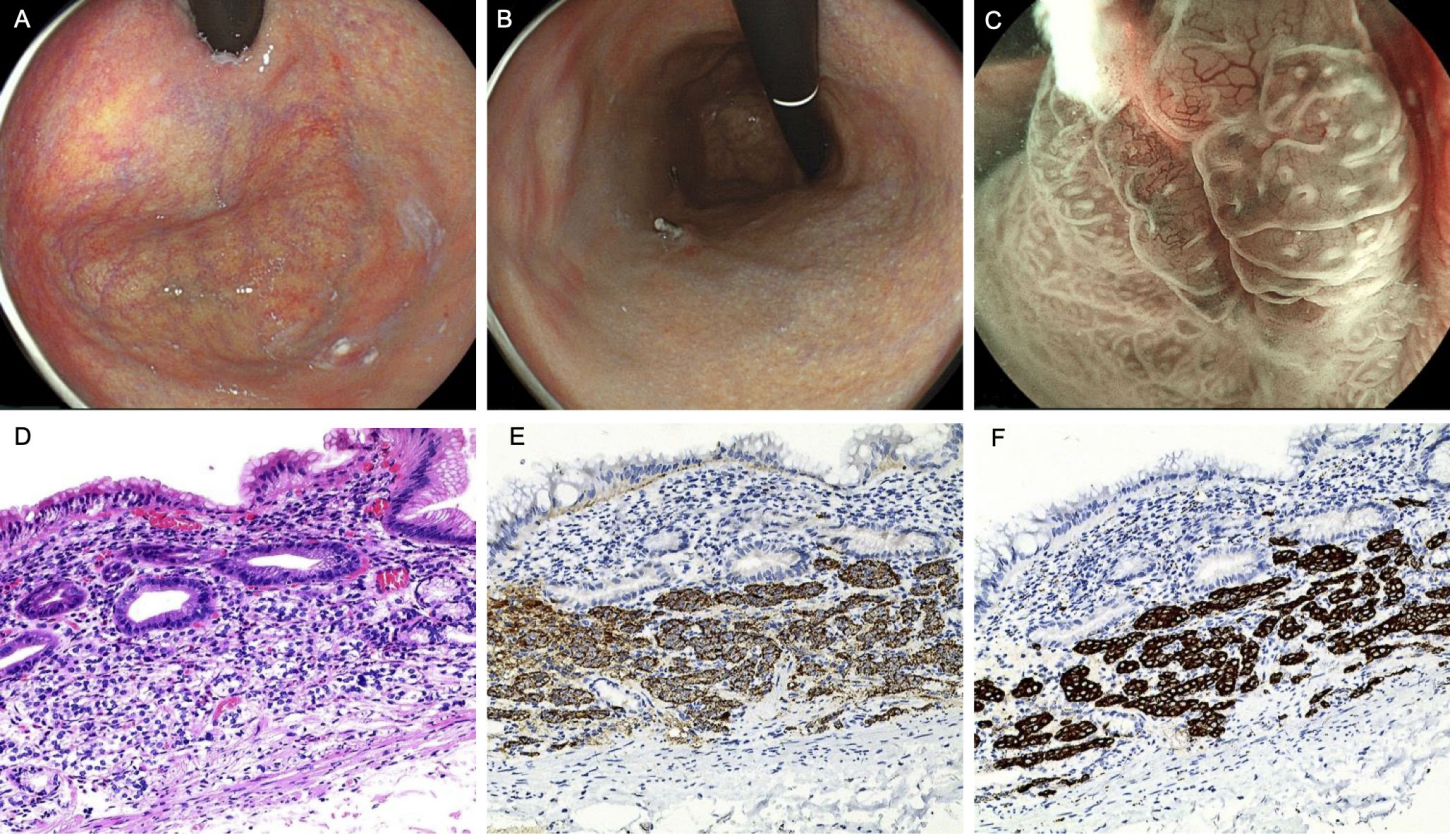
Endoscopic and pathological findings in Case 2. **(A)** Atrophy of gastric fundus mucosa with sticky adherent dense mucous. **(B, C)** Thickening of the IP in the two original excised wounds, with visible large blood vessels. **(D)** the NET (G1) region (HE,×10). **(E)** Immunohistochemical staining for CgA of the NET (G1) region (×10). **(F)** Immunohistochemical staining for Syn of the NET (G1) region (×10).

A 56-year-old woman with a 30-year history of upper abdominal discomfort underwent gastroscopy at a local hospital, which identified two bulging lesions in the gastric corpus. The lesions were resected via EMR and postoperative pathology confirmed NET. After referral to our hospital, magnifying chromoendoscopy revealed severe atrophic gastritis with mucosal edema and erosion involving the fundus and corpus, and sticky adherent dense mucous in adjacent mucosa (**Figure 2A**). The lesions sites showed thickened IP and prominent blood vessels at the base of titanium clips, indicating residual NET (**Figures 2B, C**). Subsequent ESD was performed on the two corpus lesions. Histopathological evaluation confirmed NET (G1) and revealed H. pylori infection (**Figures 2D–F**).

Laboratory findings showed reduced hemoglobin levels, with decreased serum iron and ferritin, while folate and vitamin B12 levels remained normal. IFA were negative, but PCA tested positive. The level of PG-I and the ratio of PG-I/PG-II decreased, while gastrin levels remained normal. Thyroid peroxidase antibodies were elevated and the patient had a history of hyperthyroidism.

In conclusion, the patient was diagnosed with AIG combined with H. pylori-associated gastritis, complicated by multifocal gastric NETs. Postoperatively, she was treated with oral iron supplementation, resulting in anemia correction and normalization of ferritin levels. Follow-up endoscopy showed no evidence of tumor recurrence.

### 2.3 Case 3

**Figure 3:**
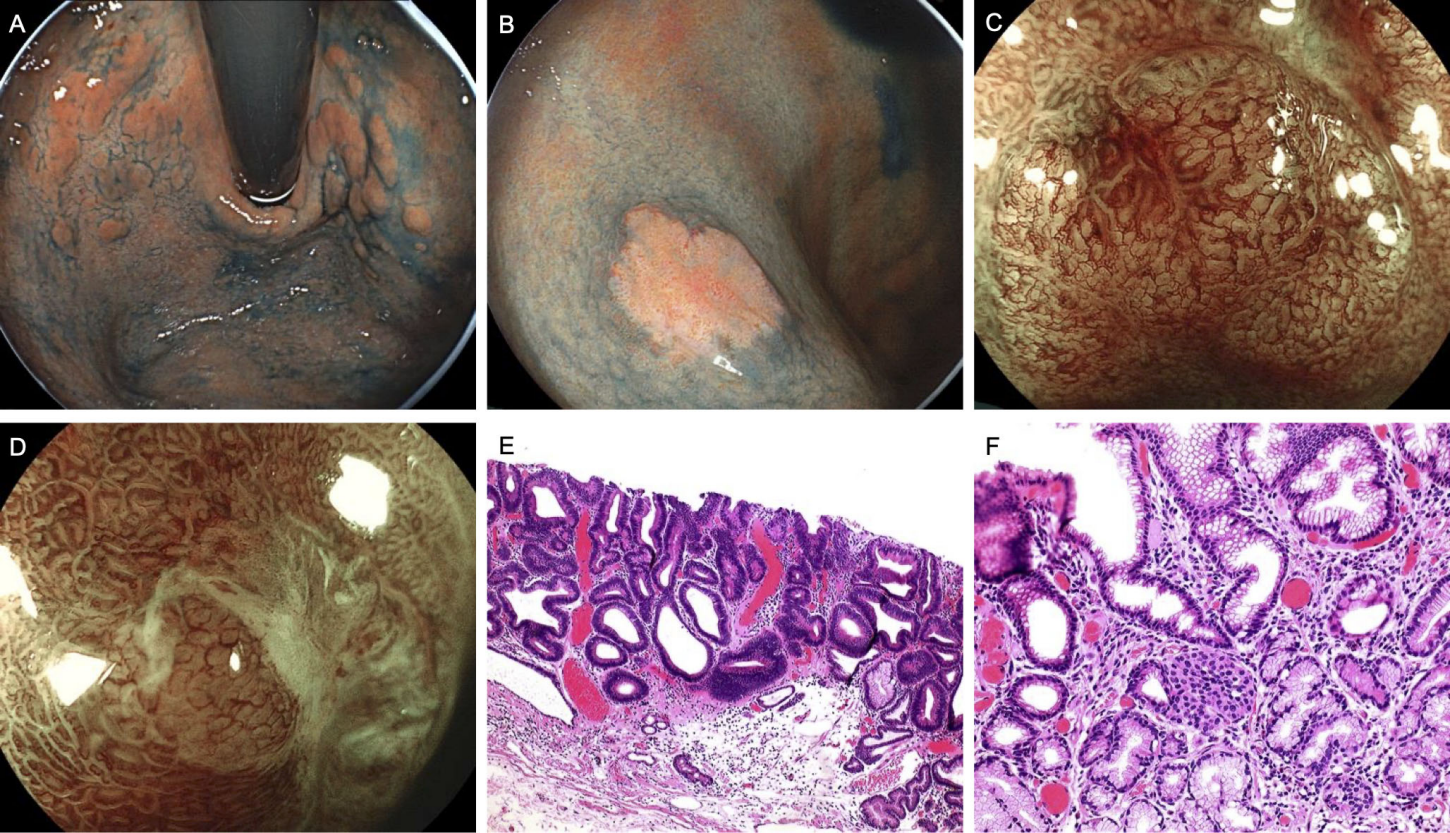
Endoscopic and pathological findings in Case 3. **(A)** Remnant oxyntic mucosa. **(B, C)** Lesions on the proximal posterior wall at the side of the greater curvature in the middle part of the gastric corpus were observed with LCI and ME-BLI. **(D)** ME-BLI observation of lesions in the anterior wall of the gastric corpus. **(E)** Well-differentiated tubular adenocarcinoma: The tumor exhibits well-formed glandular structures with clear lumina, resembling normal gastric glands. Tumor cells show abundant eosinophilic cytoplasm, round to oval nuclei with fine chromatin, and inconspicuous nucleoli. (HE,×4). **(F)** Proliferation of small foci of neuroendocrine cell nests. (HE,×10).

A 52-year-old woman presented for evaluation with gastroscopy findings indicating severe atrophy of the gastric fundus and corpus, with remnant oxyntic mucosa in the corpus region (**Figure 3A**). Magnifying endoscopic blue laser imaging (ME-BLI) identified a 1.2 cm flat elevated lesion on the posterior wall of the gastric corpus. The lesion exhibited a slightly yellowish surface with well-demarcated borders. Glandular ducts appeared morphologically regular, whereas surface microvessels demonstrated a thickened, lattice-like architecture with partial disorganization (**Figures 3B, C**). An additional lesion, a 0.5 cm erosion on the anterior wall of the gastric corpus, presented as a slightly concave area covered with a white moss-like exudate (**Figure 3D**). ME-BLI revealed sharply defined lesion borders. However, the glandular structures and portions of the surface microvasculature appear indistinct. Histopathological evaluation following ESD confirmed the presence of H. pylori infection. The posterior wall lesion was identified as a well-differentiated tubular adenocarcinoma (**Figure 3E**), while the anterior wall lesion was characterized as a NET, composed of small nests of neoplastic neuroendocrine cells (**Figure 3F**).

Laboratory investigations revealed hypergastrinemia, with decreased levels of PG-I and a reduced PG-I/PG-II ratio. IFA were negative, while PCA tested positive. The level of Vitamin B12 was decreased, while folate, serum iron, and hemoglobin levels were normal. Notably, the patient had been diagnosed with gastric corpus atrophy five years prior, during which H. pylori was not detected.

Based on these findings, the patient was diagnosed with AIG concomitant with H. pylori-associated gastritis, which had progressed to the development of a well-differentiated tubular adenocarcinoma and a NET. After vitamin B12 supplementation, serum levels normalized, and no evidence of tumor recurrence was observed on follow-up endoscopy.

### 2.4 Case 4

**Figure 4:**
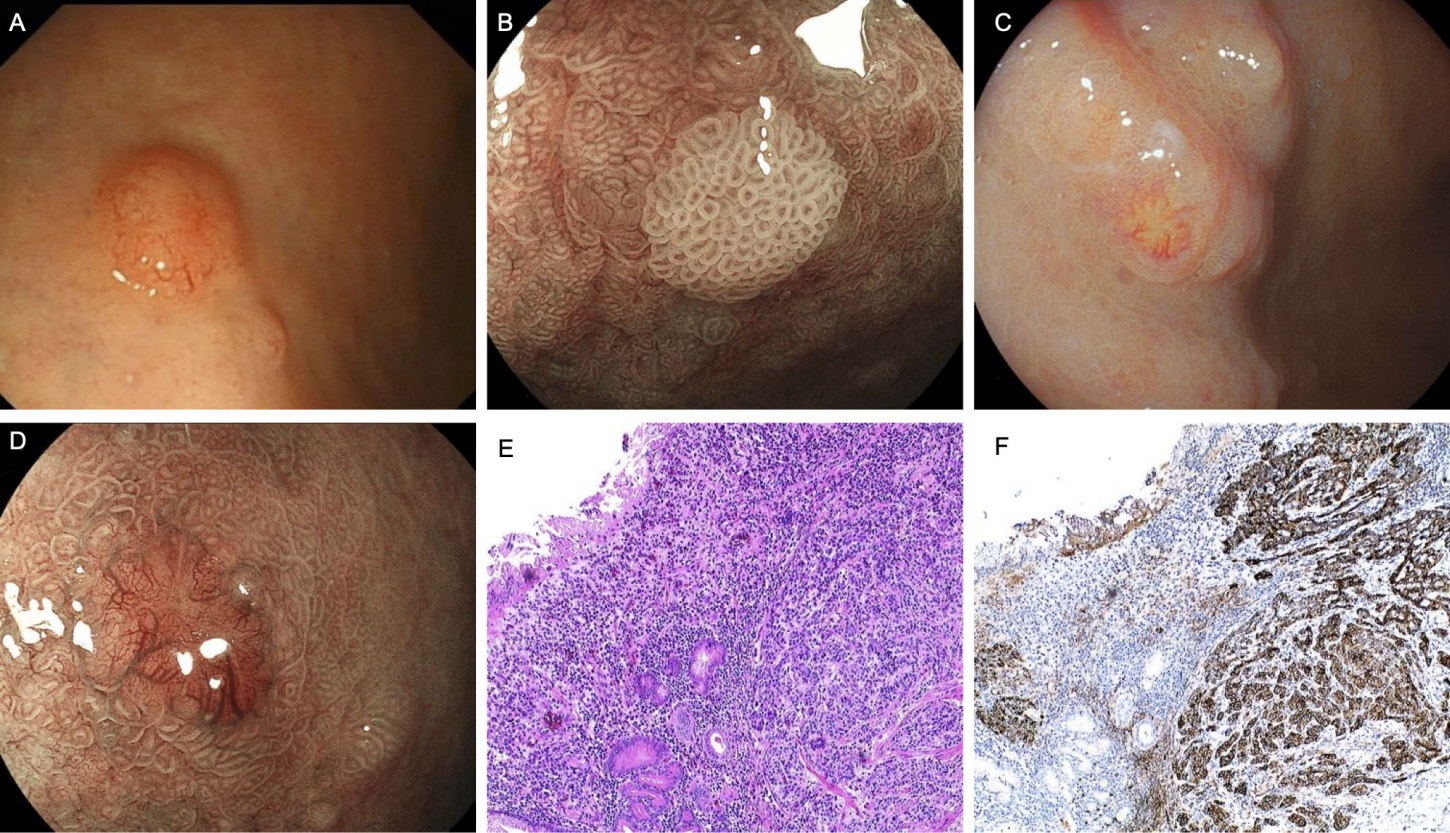
Endoscopic and pathological findings in Case 4. **(A)** Neuroendocrine tumor was found under gastroscopy two years ago. **(B)** Multiple polyps, the glandular ducts are still regular on magnification. **(C, D)** A bulging lesion in the greater curvature of the corpus with a slightly reddened surface. **(E)** Neuroendocrine cell proliferation (HE,×10). **(F)** Immunohistochemical staining for CgA of the NET (G1) region (×10).

A 75-year-old male presented with an initial complaint of indigestion two years ago. Gastroscopy identified a bulging lesion in the gastric corpus, which was resected. Pathological analysis confirmed the lesion as a Grade 1 NET, and the H. pylori test was negative (**Figure 4A**). One year later, the patient developed megaloblastic anemia, which was diagnosed through bone marrow aspiration. Despite regular intramuscular vitamin B12 supplementation, the patient recently experienced worsening dyspeptic symptoms, accompanied by weight loss and fatigue, raising clinical suspicion of AIG. Magnified staining endoscopy revealed severe mucosal atrophy multiple small polyps (**Figure 4B**), and a 0.3 cm reddish bulging lesion in the gastric corpus. ME-BLI highlighted prominent dark green blood vessels associated with the lesion (**Figures 4C, D**). Pathological examination confirmed this lesion as H. pylori-positive and a Grade 1 NET (**Figures 4E, F**).

Serological tests showed positive IFA, negative PCA, elevated gastrin levels, reduced PG-I, and a decreased PG-I/PG-II ratio. Vitamin B12 remained low, while folate, serum iron, and hemoglobin levels were normal.

Based on clinical history and test results, the patient was diagnosed with AIG complicated by Hp-associated gastritis, with the development of NET. After continued intramuscular vitamin B12 supplementation, vitamin B12 levels normalized. Follow-up endoscopy revealed no evidence of new NET lesions.

## 3 Discussion

AIG typically develops in adulthood and progresses more rapidly than H. pylori-associated gastritis. Endoscopically, AIG is characterized by atrophy predominantly affecting the gastric fundus and body, often referred to as inverse atrophy Common findings include sticky adherent dense mucus, remnant oxyntic mucosa, white globe appearance, and cast-off skin appearance (9). Disease progression can result in complete atrophy of acid-secreting glands due to autoimmune destruction of parietal cells and proton pumps. This reduces gastric acid secretion and weakens the inhibitory effect on antral G cells, resulting in compensatory G-cell hyperplasia and marked secondary hypergastrinemia. Serologically, AIG patients typically exhibit decreased levels of PG-I and a reduced PG-I/PG-II ratio, reflecting the selective loss of PG-I-secreting oxyntic mucosa. Positive IFA or PCA further support the diagnosis (10). However, the epidemiology and prevalence of AIG remain unclear, and current prevalence data may be underestimated. Contributing factors include the asymptomatic or mild presentation of early AIG, low screening rates, insufficient biopsies, and inadequate follow-up in patients treated for anemia with iron or vitamin B12.AIG is often associated with other organ-specific autoimmune diseases. A retrospective study by Kalkan et al. involving 320 AIG patients found that 53.4% had comorbid autoimmune diseases, with autoimmune thyroiditis being the most common (11). In our series, two of the four patients (Case 1 and Case 2) had a history of thyroid disease, consistent with these findings.

In contrast, H. pylori-associated gastritis predominantly affects the antrum, sparing oxyntic mucosa, which retains some gastric acid production and hypergastrinemia is often less pronounced. This may be related to antral atrophy impairing G-cell-mediated gastrin secretion (12).

The diagnosis of AIG requires a comprehensive evaluation, including endoscopic findings, the presence of autoantibodies, elevated serum gastrin levels, and characteristic histopathological changes, such as parietal cell destruction or loss, pseudopyloric metaplasia, intestinal metaplasia, and G-cell and enterochromaffin-like (ECL) cell hyperplasia (13). In our report, all four patients showed gastric mucosal atrophy involving the fundus and body, with mucosal thinning, loss of rugal folds, and visible submucosal vessels on endoscopy. Laboratory results revealed positivity for one or more antibodies (PCA and/or IFA), decreased PG-I levels, and reduced PG-I/PG-II ratios in all cases. Combined with histopathological findings, the diagnosis of AIG with concurrent H. pylori-associated gastritis was confirmed.

AIG has been linked to an increased risk of NETs due to hypergastrinemia-induced ECL cell hyperplasia. When hyperplastic ECL cell nests exceed 0.5 mm in diameter or infiltrate the submucosa, NETs can be diagnosed. In patients presenting with multiple gastric NETs, differential diagnosis with multiple endocrine neoplasia type 1 (MEN1) syndrome should also be considered. MEN1 typically involves parathyroid hyperplasia, pituitary adenomas, and pancreatic NETs, and often presents in younger individual (14). Given that all patients in this report were over 50 years old and exhibited no MEN1-related clinical manifestations, family history, or other endocrine abnormalities, MEN1 is not considered in these cases. However, as genetic testing was not performed, MEN1 cannot be entirely excluded theoretically. Therefore, MEN1 genetic screening is recommended for younger patients or those with a suggestive family history to rule out potential hereditary syndromes.

A prospective study by Massimo Rugge followed 211 H. pylori-negative AIG patients for a cumulative 10,541 person-years without observing a significantly increased risk of gastric cancer (GC). The study suggested that previously undetected H. pylori infections might contribute to the GC risk in AIG patients (15). Nguyen’s AIG mouse study has demonstrated that AIG-associated inflammation induces pathological and molecular changes linked to GC, including gastric intraepithelial neoplasia and dysplasia (16). Additionally, some studies suggest that H. pylori infections may contribute to AIG development. For instance, molecular mimicry and cross-reactivity between H. pylori-induced antibodies and parietal cell components may trigger autoimmune responses (17–20). Ihara et al. reported two cases where H. pylori-associated gastritis progressed to AIG over several years without eradication therapy (21). Another case showed rapid AIG progression after H. pylori eradication (22), suggesting complex interactions between H. pylori and AIG. However, contrasting findings indicate that PCA-mediated AIG and H. pylori infection may involve independent pathways (23, 24). A recent study demonstrated that gastric metaplasia induced by H. pylori infection and AIG exhibits highly similar histological and transcriptomic features. Both conditions were found to express a metaplastic subtype with carcinogenic potential, characterized by the expression of the cancer-associated marker ANPEP/CD13. These findings suggest that both Hp infection and AIG can drive pathological changes associated with gastric carcinogenesis (25). In our series, two patients (Case 3 and Case 4) were H. pylori-negative at the time of diagnosis. One patient presented with advanced gastric atrophy during their initial endoscopy, while the other exhibited megaloblastic anemia. These findings suggest that AIG in these patients may have preceded H. pylori infection. Further research is needed to clarify the immunopathological mechanisms and interplay between AIG and H. pylori.

Our case series differs from prior literature by documenting tumor development in the unique context of AIG coexisting with H. pylori-induced gastritis. This dual chronic inflammation may create a distinct tumorigenic microenvironment not fully addressed in previous studies. In our report, all four patients had multiple polyps or elevated lesions predominantly in the gastric fundus and body. Notably, one patient exhibited four distinct histological subtypes of gastric cancer within a single lesion, while two patients developed multifocal NETs, and one presented with well-differentiated adenocarcinoma coexisting with NETs. All tumors were located in the middle to upper stomach, and all patients were H. pylori-positive. All patients denied smoking or alcohol consumption and had no known family history of gastric cancer, suggesting that these neoplastic changes may be driven by the interaction between autoimmune gastritis (AIG) and H. pylori-associated gastritis, rather than traditional risk factors.

These findings underscore the importance of routine surveillance strategies specifically in patients with AIG concomitant with H. pylori-associated gastritis. Current guidelines offer significantly different recommendations: the European Society of Gastrointestinal Endoscopy (ESGE) suggests endoscopic surveillance every 3 years (26), while the American College of Gastroenterology (ACG) advocates symptom-driven endoscopy (27). In our cohort, two patients (Cases 1 and 3) were diagnosed with asymptomatic tumors during routine endoscopic examination, supporting the need for a more proactive surveillance approach. Based on these observations, we recommend that patients with AIG complicated by H. pylori-associated gastritis undergo chromoendoscopy with magnification and targeted biopsies every 6–12 months, together with monitoring of serum gastrin and chromogranin A levels. In addition, regular assessment of vitamin B12 and iron status is advised, as deficiencies in these nutrients may precede malignant transformation and serve as early clinical indicators.

## 4 Conclusion

Based on the four cases reported in this study and literature analysis, we propose that proactive surveillance, dynamic evaluation of gastric mucosal changes, and timely implementation of appropriate interventions are essential to mitigate the risk of gastric cancer in these patients. The coexistence of AIG and H. pylori-associated gastritis may accelerate gastric tumorigenesis through mechanisms such as exacerbation of mucosal inflammation and activation of oncogenic pathways. However, whether H. pylori infection significantly increases cancer risk in individuals with AIG remains inconclusive. Further large-scale, multicenter clinical and experimental studies are required to elucidate the impact of H. pylori infection on gastric cancer development in AIG patients and to evaluate the efficacy of therapeutic interventions. We recommend proactive surveillance and comprehensive autoimmune and nutritional evaluation in these patients to facilitate earlier diagnosis and potentially prevent malignant progression.

## Data Availability

The raw data supporting the conclusions of this article will be made available by the authors, without undue reservation.
